# Antioxidant, Immunostimulant, and Growth-Promoting Effects of Dietary *Annona squamosa* Leaf Extract on Nile Tilapia, *Oreochromis niloticus*, and Its Tolerance to Thermal Stress and *Aeromonas sobria* Infection

**DOI:** 10.3390/ani13040746

**Published:** 2023-02-19

**Authors:** Salem Hamad Almarri, Alshimaa A. Khalil, Abdallah Tageldein Mansour, Walaa El-Houseiny

**Affiliations:** 1 Fish and Animal Production and Aquaculture Department, College of Agriculture and Food Sciences, King Faisal University, P.O. Box 420, Al-Ahsa 31982, Saudi Arabia; 2 Department of Aquatic Animal Medicine, Faculty of Veterinary Medicine, Zagazig University, Zagazig 44511, Egypt; 3 Fish and Animal Production Department, Faculty of Agriculture (Saba Basha), Alexandria University, Alexandria 21531, Egypt

**Keywords:** *Annona squamosa* extract, redox status, immune stimulant, growth, bacterial challenge, stress tolerance

## Abstract

**Simple Summary:**

Custard apple (Annona squamosa) leaf extract (ASLE) is a phenolic-rich substance and considered a non-conventional feed additive. The present study aimed to evaluate the effect of ASLE dietary supplementation in the diet of Nile tilapia on growth performance, physiological status, and stress resistance. The results revealed that dietary ASLE, up to a 20 g/kg diet, improved growth and feed utilization. ASLE improved the hematological parameters, vital organ functions, and redox status in the fish. Furthermore, increasing ASLE dietary levels improved fish resistance against *Aeromonas sobria* challenge. Therefore, ASLE could be a potential feed additive in Nile tilapia diets.

**Abstract:**

Plant extracts are a phytochemically-rich alternative to antibiotic and synthetic feed additives, with high systemic bioactivity in animals. The present study aimed to evaluate the effect of a hydroalcoholic extract of custard apple (*Annona squamosa*) leaf (ASLE) on the growth, hematobiochemical parameters, digestive enzyme activities, redox status, nonspecific immune response, and cold and bacterial infection tolerance in Nile tilapia (*Oreochromis niloticus*). A total of 300 Nile tilapia fingerlings (11.87 ± 0.48 g) were fed ASLE-supplemented diets at increasing levels of 0, 5, 10, 15, and 20 g/kg for 60 days. At the end of the feeding period, the fish were experimentally challenged with cold water stress or *Aeromonas sobria*, and mortalities were recorded for 10 days. The results revealed that the growth performance and feed conversion ratio were significantly improved with an increasing level of ASLE supplementation. The hematologic profile and hepato-renal functions were retained within a healthy range in the various groups supplemented with an ASLE diet. Antioxidant status was significantly improved in the serum of fish fed ASLE-supplemented diets, in terms of superoxide dismutase (SOD), catalase (CAT) activities, reduced glutathione, and total antioxidant capacity. Meanwhile, the myeloperoxidase (MPO) and malondialdehyde (MDA) levels decreased significantly. Similarly, there was a noticeable improvement in the hepatic CAT and SOD activities and a reduction of hepatic MDA. Marked improvements in lysozyme activity, nitric oxide production, complement3 level, and phagocytic activity were recorded in groups fed ASLE-supplemented diets, which peaked with the 20 g ASLE/kg diet. Moreover, the serum glucose and cortisol levels significantly declined in groups fed ASLE at levels of 15–20 g/kg compared to the other groups. Supplementation with ASLE increased the activities of protease, lipase, and α-amylase. ASLE supplementation at a concentration of 10–20 g/kg diet enhanced the resistance of Nile tilapia to *A. sobria* infection. According to this study, ASLE supplementation enhanced the antioxidant balance, non-specific immune response, physiological status, resistance against infection, and growth performance of Nile tilapia at supplementation levels of 10–20 g/kg diet.

## 1. Introduction

Aquaculture plays, and will continue to play, a crucial role in expanding global fish production to meet the growing demand for aquatic products [1]. The most widely cultivated freshwater aquaculture species is tilapia (*Oreochromis* sp.), and it is anticipated that production will continue to increase to meet the increasing demand for fish from a population that is expanding [2,3]. Due to its fast growth, high feed utilization, natural spawning, and good edible characteristics, it is the first species raised in Egypt and the third in the world [4]. However, the expansion of aquaculture has created a number of issues, such as low growth, poor health, and increased susceptibility to infectious diseases. These diseases, including bacterial diseases, are opportunistic and can have a significant negative economic impact on freshwater and marine aquaculture [5,6]. 

In addition, rapid climate change possess a significant threat to ecosystems and living things, notably fish as ecthothermic animals, for which water temperature is an important environmental factor. Moreover, fish are regularly exposed to a variety of stresses in their intense production system [7]. Conditions of temperature stress may result in disruption of the physiological balance of the organism and subsequently hinder growth and survival [8]. Therefore, a significant challenge is faced by fish farmers and the aquaculture industry to enhance culture performance and combat stress.

The use of conventional medications and vaccines for disease prevention and treatment of disease comes with significant drawbacks [9]. In addition, the use of antibiotics in aquaculture to treat and prevent bacterial infections may result in bacteria that are resistant to the antibiotics or the presence of antibiotic residues in fish raised for human consumption [10]. As a result, there is a crucial need to create safe solutions, as affordable alternatives to conventional methods of disease control, to maintain an eco-friendly aquaculture. Over time, there has been significant progress in fish nutrition, which has led to the creation of specialized feed formulations and novel, balanced commercial diets that support optimum growth and the production of high-quality, healthy fish [11]. Various efforts have been made to use medical and aromatic herbs as feed additives, which are innovative approaches to reducing disease risk, as well as boosting the immune system during exposure to stressors such as rough handling, transport, cold temperature, and poor water quality for reared fish [12,13,14]. Among these herbs is Custard Apple, *Annona squamosa*, which is a member of the Annonaceae family and a species of Annona, which is known mainly for its edible fruits [15]. Various parts of *A. squamosa* are used in folkloric medicine to treat a variety of diseases [16]. Numerous active chemicals with various pharmacological properties, including anti-inflammatory and anti-tumor actions, were identified in phytochemical analyses [17]. It is used as an anti-inflammatory, anti-diabetic, hepatoprotective, cytotoxic, genetoxic, antitumor, and anti-lice agent [18]. The leaves of Custard apple contain a considerable amount of phenol-based compounds, mainly alkaloids and flavonoids [19,20]. Leaf extracts produced with various solvents were shown to have antibacterial action and they also included sterols, flavonoids, and tannins [21]. Custard apple (*Annona squamosa*) leaf extract (ASLE) has health-promoting effects and can be used as a potential active ingredient in drugs and functional foods [19]. ASLE had immunostimulatory effects in Catfish [15] and increased resistance to *Aeromonas hydrophila* [22]. In addition, studies on other animal species and invitro experiments showed anticancer, antidiabetic, antioxidant, antimicrobial, antiobesity, lipid-lowering, and hepatoprotective functions [19,23]. 

Hence, it appears that ASLE could be beneficial for boosting immune status and lessening the susceptibility of fish to pathogens. However, its use as a dietary supplement in aquaculture has not been fully explored. Therefore, the objective of the current investigation was to evaluate the effect of dietary supplementation with various concentrations of ASLE in *O. niloticus* diets on growth, and antioxidant and immunological biomarkers, as well as to evaluate its effect on health status indicators using certain haematobiochemical indices, cold stress tolerance, and resistance to *A. sobria* infection in Nile tilapia.

## 2. Material and Methods

### 2.1. Plant Collection and Preparation

The leaves of *A. squamosa* were collected from local gardens in Sharkia, Egypt, then identified and authenticated by a botanist in the Faculty of Agriculture, Zagazig University, Egypt. The leaves were properly cleaned with distilled water after being washed with tap water, then dried at 45 °C. In an electronic grinder, the plant material was crushed and ground into a fine powder before being stored in an airtight plastic container for further utilization. The leaves extracts were obtained as follows: in a shaking water bath at room temperature for 30 min, 6 g of plant powder was extracted using 100 mL of ethanol:distilled water (1:1). The extract supernatants were collected and filtered using Whatman paper after centrifugation for 15 min at 3000 rpm. The solvent was finally evaporated by vacuum evaporation through a rotary evaporator. The dried extract was maintained in a refrigerator at 4 °C [24]. The bioactive components present in ASLE used in the present study were identified through gas chromatography-mass spectroscopy (GC–MS) analysis [25]. The dominant compounds were sodium benzoate (27.50%), 4,4-Tert-Butylcalix (4) arene (12.34%), 4,4-Dimethylcholesterol (10.30%), Butyloctylpthalate (9.67%), stigmasterol acetate (2.92%), and isoamylacetyate (2.29%).

### 2.2. Fish Rearing Conditions 

Healthy Nile tilapia (*O. niloticus*) (N = 300, mean weight 11.87 ± 0.48 g) were obtained from nursery ponds at the Central Laboratory for Aquaculture Research (CLAR), Abbassa, Sharkia Province, Egypt. The fish were free of any history of disease or outbreaks. Prior to the experiment, a routine check of the fish’s health was performed [26]. The fish were kept in 80 L capacity glass aquaria and filled with chlorine-free tap water with continuous air supply. The fish were acclimatized to the experimental conditions for 15 days prior to the start of the experiment and fed by hand with a basal diet up to satiation. The light–dark cycle was kept at 12 h/12 h. To maintain water parameters within the optimal recommended levels for growth and survival during the period of the experiment, dissolved oxygen, water temperature, and pH were monitored daily. The water temperature ranged from 25.85 to 26.6 °C, dissolved oxygen was 6.3–6.9 mg/L, pH was 7.2–7.7, and ammonia concentration was 0.20 to 0.25 mg/L. All levels were within the permitted limits for fish aquaculture [27]. Twice a week, three quarters of the water in the aquarium was drained along with settled fish excrement and refilled with fresh and aerated water from a holding tank. The Institutional Animal Care and Use Committee of Egypt’s Zagazig University accepted the experimental protocol (approval no. ZU-IACUC/2/F/284/2022).

### 2.3. Experimental Design and Diets

Nile tilapia were randomly divided into five quadruple groups (15 fish/replicate, N = 60 fish/group). Five experimental diets were designed by adding the extract of the ASLE to the formula at levels of 0 g/kg diet (control), 5 g/kg diet (0.5%), 10 g/kg diet (1%), 15 g/kg diet (1.5%), and 20 g/kg diet (2%). The ingredients of diets were mixed mechanically, forming pellets of 1.5 mm diameter using a pellet machine. The prepared diets were air-dried for 24 h at room temperature and stored in a refrigerator at 4 °C until use. The chemical composition and ingredients of the basal and experimental diets are shown in Table 1.

The experimental diets were offered to the fish at a rate of 3% of their total biomass two times per day (8:00 a.m. and 3:00 p.m.) for 60 days. In accordance with the change in fish weight, the introduced diet % was modified every two weeks, and the uneaten diet was collected, dried, and subtracted from the introduced feed for accurate calculation of feed intake. 

### 2.4. Growth Performance

The fish were sampled every two weeks, to assess growth performance using a sensitive weight balance. The final weight, weight gain (%), specific growth, and feed conversion rate were determined as follows:

Specific growth rate (SGR) = 100 × (Lin W2 − Lin W1)/days

where W2 = Weight of fish at time T2 (final), W1 = Weight of fish at time T1 (initial)

Weight gain (%) = 100 × (weight gain/initial weight)

Feed intake (FI) = Feed consumed/Number of surviving fish

Feed conversion rate (FCR) = Total feed consumed by fish (g)/Weight gain by fish (g)

Protein productive value (PER)= weight gain (g)/protein intake (g)

### 2.5. Sampling

At the end of the feeding experiment (60 days), blood samples were taken from four randomly chosen fish from each aquarium (16 fish per group), after being anesthetized with 95 mg L^–1^ clove oil (Oleum Cosmetics, Cairo, Egypt) [28]. From the caudal vessels, blood was drawn in vials containing heparin for hematological parameters. Sterile syringes without anticoagulants were used to collect other blood samples and centrifuged at 1075× *g* for 20 min, to obtain serum samples and stored in a deep freezer at −20 °C until use. The serum samples were used to perform the biochemical and immunological assays. 

Following blood sampling, the fish were necropsied aseptically and samples of the liver and intestines were retrieved. These tissues were preserved on ice-cold dishes, cleansed with ice-cold sterile saline, dried with filter paper, split, and then frozen at −20 °C. Following that, 100 mg of each tissue was added to a tube containing 1 mL of a buffer (10 mM phosphate/20 mM tris-pH 7.0) and homogenized using a Teflon homogenizer, then centrifuged at 10,000× *g* for 5 min at 4 °C. The supernatants were pooled after centrifugation and kept at −80 °C until use. Intestinal homogenates were used to assess the activity of digesting enzymes, while liver homogenates were employed to identify markers of oxidative stress.

### 2.6. Evaluation of Health-Related Parameters

#### 2.6.1. Hematological Analyses

Blood parameters assayed were red blood cells (RBC) and differential white blood (WBC) cell counts, using an improved Neubauer hemocytometer with Natt and Herrick diluting fluid. Hemoglobin (Hb), packed cell volume (PCV), mean corpuscular volume (MCV), and mean corpuscular hemoglobin (MCH) were immediately measured after sampling, according to the methods described by Jain [29].

#### 2.6.2. Hepatorenal Function Indicators and Stress Indicators

Serum total proteins and albumin were spectrophotometrically evaluated using techniques described by Henry [30] and Reinhold [31], respectively. Furthermore, serum globulins were calculated using the method of Coles [32]. Aspartate aminotransferase (AST), alkaline phosphatase (ALP), alanine aminotransferase (ALT), creatinine, and urea were evaluated in serum using Spinreact kits (Esteve De Bas, Girona, Spain), according to the protocols established by Wenger et al. [33], Burtis and Ashwood [34], Murray [35], Fossati et al. [36], and Kaplan [37], respectively. The serum glucose was estimated using colorimetric diagnostic kits from spectrum-bioscience (Egyptian Company for Biotechnology, Cairo, Egypt) using the techniques of Trinder [38]. The serum cortisol level was evaluated following the method outlined by Tunn, et al. [39].

#### 2.6.3. Assessment of Oxidant/Antioxidant Status

Antioxidant activities in serum and liver homogenate were evaluated using colorimetric commercial kits purchased from Biodiagnostic Co., Cairo, Egypt. Total antioxidant capacity (TAC) was estimated in serum using colorimetric commercial kits purchased from Bio-Diagnostic Co. (Cairo, Egypt) [40]. Catalase (CAT) activity was monitored using the procedure of Aebi [41]. Superoxide dismutase (SOD) activity was assessed following the protocol of Nishikimi, et al. [42]. Quantitative colorimetric glutathione dehydrogenase (GSH) was performed according to Beutler [43]. Malondialdehyde (MDA) was monitored using the technique of Uchiyama and Mihara [44]. The activity of myeloperoxidase (MPO) in fish serum was measured using the approach of Kumari and Sahoo [45].

#### 2.6.4. Non-Specific Immunological Assessment

Serum lysozyme activity was determined using the turbidimetric method [46] with a suspension of *Micrococcus lysodeikticus* (Sigma-Aldrich, Burlington, MA, USA). This test is based on the lysis of a Gram-positive bacterium that is sensitive to the lysozyme (*M. lysodeikticus*). Nitric oxide (NO) and complement3 (C3) levels were determined using ELISA kits (MyBioSource, San Diego, CA, USA), according to the manufacturer’s instructions. The phagocytic activity (%) of leucocytes was assayed in heparinized blood according to Siwicki, et al. [47]. Phagocytic activity = (number of phagocytic cells that phagocytise bacteria/total number of phagocytic cells counted) × 100.

#### 2.6.5. Digestive Enzyme Assays in the Intestine

Following the manufacturer’s instructions, commercial colorimetric kits (Biodiagnostic Co., Cairo, Egypt) were used to measure the activity of protease, α-amylase, and lipase enzymes in intestinal homogenates from the various groups.

### 2.7. Challenge with Cold Temperature Stress

After the feeding period, fish from each treatment were randomly deposited in triplicates at a rate of five fish per replicate. Using a thermostat attached to the cooling system, the fish were gradually exposed to cold water. The temperature started at 25 °C and was subsequently reduced by one degree every 12 h by regulating the thermostat until reaching 18 °C. Nile tilapia showed severe growth retardation, decreased antioxidant enzyme activities, and immunosuppression at 18 °C, as described by Ibrahim, et al. [14]. The temperature remained at 18 °C for 2 weeks after reaching that level. The daily fish mortality was recorded by keeping the fish under observation.

### 2.8. Aeromonas Sobria Bacterial Challenge

At the end of the test period (60 days), five fish per replicate (N = 20 fish/group) were challenged with *A. sobria* (previously isolated from naturally infected Nile tilapia in the Department of Aquatic Animal Medicine, identified and confirmed to be pathogenic). At the Department of Microbiology and Immunology, National Research Center (NRC), Dokki, Giza, Egypt, *A. sobria* was identified using traditional biochemical assays and an automated VITEK 2-C15 system for bacterial identification (BioMérieux, France), according to the manufacturer’s instructions and as described by Scheidegger, et al. [48] and Zhou, et al. [49]. Lethal dosage (LD50) for *A. sobria* was first recorded. A variety of doses of live bacteria were intraperitoneally (IP) injected into fish, and three days later, the infected fish mortality was observed. The LD_50_ that induced 50% fish mortality was 2 × 10^8^ CFU/mL. A sub-lethal dosage was used in the bacterial challenge test. A 0.2 mL dose of suspension cells comprising 1.5 × 10^7^/mL cells was administered intraperitoneally (IP) to the fish using standard MacFarland tubes. *A. sobria* was isolated from the dead fish to confirm responsibility for the death of the fish. The injected bacteria were re-isolated from moribund and recently dead fish and identified. For ten days, all groups were closely monitored, to note any abnormal findings and daily mortality.

### 2.9. Statistical Analysis

A one-way analysis of variance (ANOVA) was employed to conduct statistical analysis (SPSS version 16.0, SPSS Inc., Chicago, IL, USA). With statistical significance set at *p* < 0.05, the differences between groups were compared using Tukey’s multiple comparison post hoc test. The analysis findings are reported as means ± SE (standard error). Additionally, SGR and FCR were used to establish a fit regression model between the levels of ASLE and fish response.

## 3. Results 

### 3.1. Growth Performance

**Figure 1:**
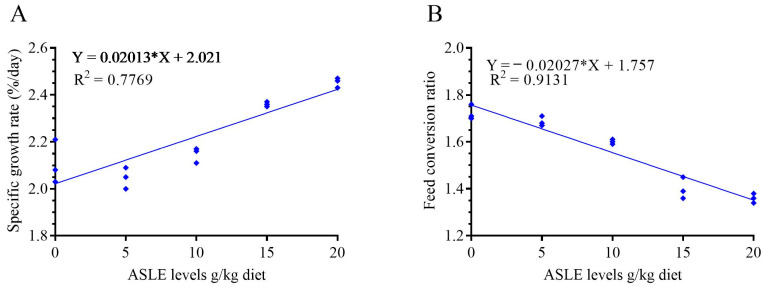
Fit linear regression model of the specific growth rate (**A**) and feed conversion ratio (**B**) in response to increasing dietary supplementation of *Annona squamosa* leaf extract (g/kg).

The effect of ASLE-supplemented diets on growth performance parameters is shown in Table 2. In general, there was a significant influence (*p* < 0.05) on all growth parameters. ASLE-supplemented diets led to a significant increase (*p* < 0.05) in the final body weight, weight gain (%), SGR, FI, and PER in ASLE_20_, followed by ASLE_15_, ASLE_10_, ASLE_5_, and the control group, respectively. The FCR showed a significant reduction in ASLE_20_, followed by other groups. The dose-response analysis revealed a substantial linear correlation between the SGR and FCR responses to increased dietary supplementation of ASLE (R^2^ = 0.83) (Figure 1). Fish survival was 100%, with no noticeable differences between the various groups of fish during the feeding period (*p* > 0.05; Table 2), and the fish in all test groups remained healthy during the feeding period based on their overall activity.

### 3.2. Hematological Indices

The hematological results of Nile tilapia fed with various ASLE levels are shown in Table 3. ASLE-fortified fish diets showed significantly improved hematologic indices (*p* < 0.05) compared to the control group. In a dose-dependent order, hematological indices, such as RBCs, Hb, PCV%, and WBC levels were markedly increased. Furthermore, the addition of ASLE to Nile tilapia diets considerably increased the values of lymphocytes, heterophils, eosinophils, and monocytes.

### 3.3. Hepatorenal Function and Stress Indicators

The serum total protein, albumin, and globulin are given in Table 4, which were enhanced (*p* < 0.05) in the ASLE_20_ and ASLE_15_ groups compared to the other groups. In the same table, the activities of ALT, AST, and ALP activities show a significant reduction (*p* < 0.05) with increasing ASLE concentration. Furthermore, the serum urea and creatinine levels were significantly decreased by increasing the ASLE level in the diets, and their lowest values were reported in the 20 ASLE g/kg diet, while the highest values were observed with the control diet. The effects of dietary ASLE on the stress indicators of Nile tilapia are illustrated in Table 4. Serum glucose and cortisol levels were significantly (*p* < 0.05) decreased in fish fed diets enriched with ASLE_20_ compared with the other groups.

### 3.4. The Activity of Antioxidant Enzymes

Dietary ASLE_20_ supplementation resulted in a significant improvement (*p* < 0.05) in the serum levels of CAT, SOD, GSH, and TAC, followed by ASLE_15_, ASLE_10_, ASLE_5_, and the control, respectively (Table 5). Conversely, the ASLE-supplemented diets reduced oxidative stress, as stipulated by the gradually declining MDA and MPO levels. The ASLE_20_ groups achieved the best results. The liver CAT and SOD activities were also considerably higher in ASLE_5_, ASLE_10_, and ASLE_15_ than in the other groups, although the concentration of MDA showed the reverse tendency (Table 5).

### 3.5. Nonspecific Immune Parameters

**Figure 2:**
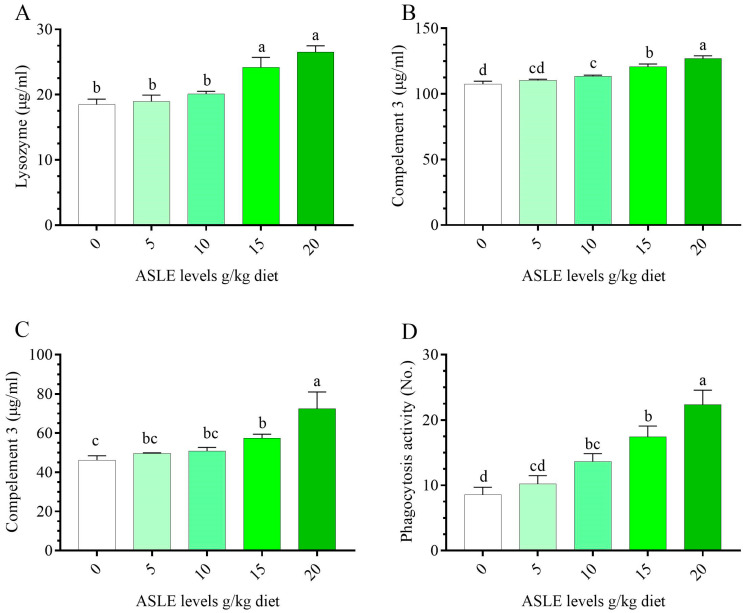
Effect of dietary supplementation with *Annona squamosa* leaf extract on innate immune parameters of *O. niloticus* for 60 days. (**A**) lysozyme, (**B**) complement 3, (**C**) nitric oxide, and (**D**) phagocytic activity. Columns bearing different letters are significantly different at *p* < 0.05.

Nile tilapia fed ASLE-based diets had a considerably higher (*p* < 0.05) non-specific immune response higher than fish fed the control diet (Figure 2). Regarding the lysozyme, and nitric oxide, C3 activities, there were significant enhancements (*p* < 0.05) observed in fish fed ASLE_20_, followed by ASLE_15_. There was a significant increase in phagocytic activity in a progressive manner in ASLE_20_, ASLE_15_, ASLE_10_, ASLE_5_, and the control. 

### 3.6. Activity of Digestive Enzymes in the Intestine

**Figure 3:**
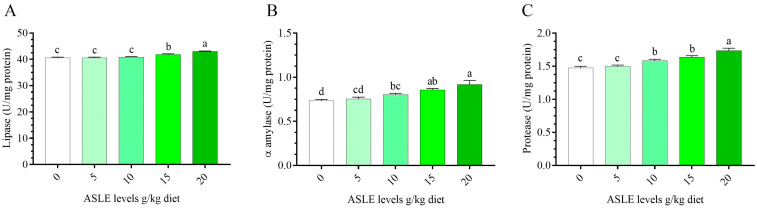
Effect of dietary supplementation with *Annona squamosa* leaf extract on intestinal digestive enzymes of *O. niloticus* for 60 days. (**A**) lipase, (**B**) α-amylase, and (**C**) protease. Columns bearing different letters are significantly different at *p* < 0.05.

Figure 3 depicts the alterations in the Nile tilapia’s intestinal digestive enzyme activity as a result of 60 days of ASLE dietary supplementation. In comparison to the control group, dietary ASLE improved the release of digestive enzymes (protease, lipase, and α-amylase), and the highest values were recorded with the 10–20 g/kg diet (*p* < 0.05).

### 3.7. Cold-Water Stress Tolerance and Challenge with A. sobria 

**Figure 4:**
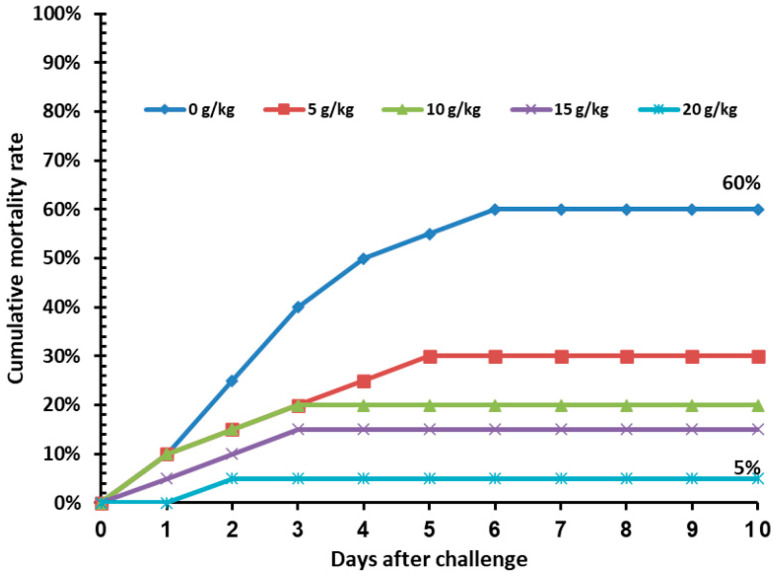
Cumulative mortality rate (%) in Nile tilapia fed increasing levels of *Annona squamosa* leaf extract for 60 days and post-challenged by *Aeromonas sobria* infection for 10 days.

Regarding cold stress, no significant (*p* > 0.05) mortality was recorded in fish under cold stress in all ASLE-supplemented treatments (Table 5). Resistance of Nile tilapia fed on ASLE-enriched diets for 60 days was recorded against the *A. sobria* challenge, in terms of percentage cumulative mortality. The control group had the highest mortality rate (60%), while the ASLE_20_-fed group had the lowest mortality rate (5%). The survival rate was increased in the fish by increasing the level of ASLE_20_ (95%), where it was 85.00%, 80.00%, and 70.00% in ASLE_15_, ASLE_10_, and ASLE_5_, respectively, as compared with the control group (40.00%), as shown in Figure 4.

## 4. Discussion 

Aquaculture provides a viable supply of affordable and healthy protein for human consumption and enhances human health [3]. Unfortunately, disease outbreaks continue to be a significant obstacle preventing advanced intensification from achieving sustainable production. Several safe and environmentally friendly compounds for modulating immune state, improve growth performance, and prevent fish disease are being investigated in aquaculture [50,51,52]. They are also used to protect aquaculture animals from external stressors such as contaminated water, cold temperatures, and overcrowding [53,54]. Therefore, our study aimed to address the potential role of ASLE in improving the health, immune, and growth performance of Nile tilapia. 

The findings of the present study demonstrated that various health indicators in Nile tilapia could be successfully improved through ASLE dietary replacement. The best results were found in the group of fish fed the 20 g ASLE/kg diet, as indicated by a significant improvement in final body weight, weight gain (%), SGR, FCR, and PER compared to fish fed the control diet. The incorporation of ASLE in the Nile tilapia diet showed statistically significant changes, which were detected in the crude protein and ash in a dose-dependent manner. These observations are consistent with those of Safira et al. [55], who investigated the effect of ASLE on *Clarias batrachus* growth rate. However, to our knowledge, no study has investigated the impact of *A. squamosa* on tilapia species. Growth enhancements can be linked with improvement of diet digestion and absorption, leading to improved nutrient utilization. This supposition was verified by the high activity of digestive enzymes observed herein. On the other hand, ASLE may reduce the number of possible pathogens in the gastrointestinal tract (Figure 4), increase the population of beneficial microorganisms, and/or increase the activity of microbial enzymes, all of which would increase feed digestibility and nutrient absorption. In addition, high concentrations of protein, fiber, carbohydrates, essential oils, vitamins, and minerals improved the nutritional status of dietary ASLE [19]. Farag & Paré [56], El-Garhy et al. [57], El-Houseiny, et al. [58]; Toutou, et al. [59] reported superior growth performance following the feeding of certain natural plants or plant extracts to Nile tilapia.

Physiological and stress conditions of fish can be evaluated using hematological parameters [60]. The current study confirmed that ASLE-fed Nile tilapia demonstrated a substantial increase in all blood indices in diets containing 20 g/kg ASLE. This finding suggests that the incorporation of ASLE into the diet could increase the immunity and prevent infection. *A. squamosa* contains a wide variety of phytochemicals, including proteins, carbohydrates, saponins, alkaloids, flavonoids, phenolics, and glycosides [61]. As ASLE has a higher protein level, both humans and animals can benefit from its nutritional value [62]. Furthermore, *A. squamosa* contains a variety of minerals, including vitamin A, C, E, B1 (thiamine), B2 (riboflavin), B3 (niacin), B9, and folic acid, that have a hematic impact. Extracts of *A. squamosa* were found to contain macro and microminerals: Mg, P, Zn, Cu, and Se [63]. To maintain general health, various minerals are necessary [64]. Similar results of enhanced hematological indices were reported with an extract of *Mitracarpus scaber* leaves being fed to Nile tilapia [65]; as well as Milk thistle and co-enzyme Q10 [58], and *A. vulgaris* powder [13].

Compared to mammals, fish have a more innate immunity for defense [66]. The phagocytic cells (neutrophils and macrophages), which are an essential part of innate immunity, play a crucial role in eliminating infections through a process known as phagocytosis. Additionally, macrophages emit a potent reactive oxygen called NO to improve their capacity to kill infections through phagocytosis [67]. Another crucial element is lysozyme, which is produced by leucocytes engaged during the start of phagocytosis and has bactericidal effects, by lysing bacterial cell walls [68]. In the current study, dietary ASLE significantly enhanced the non-specific immunological defenses in Nile tilapia compared to those in the control group in a dose-dependent manner. This may have been due to potential biological and pharmaceutical effects, including antioxidant, antibacterial, and antiviral impacts [23,69]. Other elements identified in ASLE that could help to improve fish immunity include acetogenins, alkaloids, flavonoids, phenols, saponins, tannins, glycosides, sesquiterpenes, anthocyanins, steroids, diterpenes, terpenoids, quinones, amino acids, and fatty acids [70,71]. To combat various diseases, polyphenolic chemicals play an important role in the control of a number of physiological and biochemical factors, including enzyme activity, cell differentiation, signal transduction mechanisms, and cellular redox potential [72]. Similar immunomodulatory actions were recorded in Nile tilapia with various herbal dietary additions [13,58]. 

Numerous crucial biological components, such as DNA and proteins, can be destroyed by oxidative stress. The body has a defense mechanism against oxidative damage to the tissues [73]. CAT and SOD are antioxidant enzymes that can eliminate reactive oxygen free radicals. Glutathione is a non-enzymatic antioxidant that can also counteract these radicals through enzymatic reactions. In the current study, significant increases over the control values were noted in serum antioxidant enzyme activities, including CAT, SOD, GSH, and TAC, as well as a reduction in the MDA content in the serum of fish fed ASLE-supplemented diets. The results also confirmed an improvement in the liver contents of CAT and SOD, with significant decreases in the level of MDA in ASLE-fed groups compared to the control. The leucocyte-produced MPO enzyme is a component of the innate immune system. As a physiological catalyst for lipid peroxidation, this enzyme produces ROS, which in turn affects the inflammatory response [74]. In this investigation, feeding on ASLE-supplied diets resulted in a reduction in serum MPO activity. This result may have been related to the anti-inflammatory effect of *A. squamosa*, which is related to a number of chemical compounds, including phenolics, annonaceous acetogenins, saponins, flavonoids, alkaloids, glycosides, alkaloids, steroids, and terpenoids, or due to cyclooxygenase enzyme activity inhibition, which is involved in the inflammation process [56]. The antioxidant properties of ASLE may be attributed to *A. squamosa* containing flavanoids, coumarins, alkaloids, and terpenoids [19,75,76]. Several authors have shown a significant link between fruit phenolic concentrations and antioxidant capability [18,77]. In Nile tilapia fed *A. vulgaris*, *Silybum marianum* exerted antioxidant efficacy and consequently protected tissues from oxidative stress, as stated by Mansour, et al. [13]; Khalil, et al. [78], respectively.

In this study, fish fed ASLE-supplemented diets had higher levels of total serum proteins and globulin. This finding suggests that ASLE can boost protective proteins, which can then activate the immune system. High levels of blood protein, particularly globulins, are a good predictor of enhanced liver function and innate immune response [79]. Moreover, the enhancement of serum total protein and globulin may have been due to the fact that ASLE contains a high amount of proteins and amino acids [80]. Similar results were observed in Nile tilapia fed diets supplemented with *A. vulgaris* [13].

Compounds that serve as antioxidants, lipid peroxidation inhibitors, and have the ability to scavenge free radicals may have hepato-renal protective characteristics. As we mentioned previously, ASLE has high levels of antioxidant enzyme activity. The present study suggested the idea that the incorporation of ASLE in the fish diet led to a reduction of liver functional enzymes (ALT, AST, and ALP) and kidney function indicators. Additionally, ASLE reduced MDA levels in the liver and serum. The current results were supported by Kumar, et al. [19], who mentioned that the effect of ASLE was equivalent to that of a hepatoprotective substance (silymarin), which was attributed to the abundance of coumarins, which may be the main factor causing the hepatoprotective action.

The primary indicators of fish stress are serum glucose and cortisol, which fluctuate depending on changes in the environment or in the diet. Under normal circumstances, cortisol regulates a variety of physiological processes in fish, and it also enables quick physiological changes in response to stress [81]. Several components of intermediary energy metabolism are stimulated by cortisol, which also increases oxygen absorption, boosts gluconeogenesis, and inhibits glycogen synthesis. Cortisol also appears to play a key role in both aerobic and anaerobic metabolism [82]. In the current investigation, blood glucose and cortisol levels were significantly decreased with the dietary addition of ASLE. The richness of ASLE in flavonoids and other essential minerals plays a critical role in controlling glucose uptake and lipid metabolism [19,71,83,84]. Since cortisol plays a significant role in reducing inflammation and improving immunity, as shown by the lower level of MPO in serum, ASLE is therefore believed to be particularly beneficial for reducing stress. 

Particularly during the winter months, tilapia in fish farms are occasionally exposed to sudden cold temperatures for a few hours in the early morning. Overall, the greater capacity of the tilapia supplemented with ASLE to withstand cold-water stress was a direct consequence of all of the impacts mentioned. Consistent with this result, Ibrahim, et al. [14] revealed that incorporation of rocket leaves in the diet could ameliorate tilapia fish against cold water stress.

Challenge tests are typically used as the standard assay to assess the overall health of the immune system [85]. A strong indicator of the effectiveness of immunostimulants is the increase in the resistance of fish to pathogenic microorganisms [86]. According to the findings of this study, dietary ASLE had a protective effect against *A. sobria* infection in the fish. This may have been correlated with the elevation of nonspecific immune parameters, such as phagocytic activity, lysozyme, NO, C3, and antioxidant enzyme activities, in addition to the reduced levels of glucose and cortisol, which could have strengthened the defense of fish against infection. In fact, it has also been reported to possess extraordinary pharmacological capabilities, including antibacterial action [87]. In addition, annotemoyin, an active acetogenin component obtained from chloroform leaf extract, and certain flavonoid compounds purified from the plant’s aqueous leaf extract, both demonstrated notable antibacterial properties [88]. Squamocin, squamostatin, and cholesteryl glucopyranoside are examples of other acetogenins that have been shown to suppress the growth of certain Gram-positive and Gram-negative bacteria [18,89]. The primary components of ASLE’s antimicrobial mechanism of action are the phenolic compounds, which disrupt bacterial metabolic processes, cause cytoplasmic component coagulation and leakage, and have an anti-quorum sensing function [90]. Our findings agree with those of Paul, et al. [55], who investigated the impact of ASLE on the survival rates of *C. batrachus.* The inclusion of phytochemical components and the antimicrobial characteristics of ASLE contributed to the higher survival rates. The enhancement in the disease resistance of Nile tilapia has been reported when fed various medicinal plants or their extracts [13,91,92]. 

## 5. Conclusions 

The findings of the current investigation demonstrated that the dietary addition of ASLE at a dose of 20 g/kg could improve the measured blood parameters, demonstrating its hemostatic efficacy on RBCs, Hb, PCV, and WBCs, as well as the immune response, antioxidant status, and tolerance of Nile tilapia to cold water stress and *A. sobria* infection. Improvements in hepatorenal function, immunological, and antioxidant parameters may strengthen fish’s ability to fight off diseases and tolerate stress, which would ultimately benefit the aquaculture sector. However, more research is required to determine the molecular, immunomodulatory, and other effects brought about by ASLE in other species of fish.

## Figures and Tables

**Table 1:** Ingredients and proximate chemical analysis of the experimental diets (g/kg).

Ingredients (g kg^−1^)	ASLE Levels g/kg Diet				
	0	5	10	15	20
Hearing fish meal (65% protein and 9% fat)	110	110	110	110	110
Soybean meal (44% protein and 1.9% fat)	420	420	420	420	420
Ground corn (8% protein and 2% fat)	290	285	280	275	270
Wheat bran (12% protein and 0.2% fat)	100	100	100	100	100
Corn oil	30	30	30	30	30
Cod liver oil	20	20	20	20	20
ASLE	0	5	10	15	20
Vitamin premix ^1^	10	10	10	10	10
Mineral premix ^2^	20	20	20	20	20
Chemical analysis					
Crude protein (N × 6.25)	308	305	307	308	309
Crude lipids	73	73	77	77	77
Crude fiber	53	53	55	57	57
Ash	72	72	73	74	74
Nitrogen free extract ^3^	494	497	488	484	483
Gross energy (kcal/kg) ^4^	446.03	445.57	446.78	445.70	445.86

^1^ Vitamin pre-mix (per kg of pre-mix): vitamin A, 88,000 IU; vitamin E, 7000 mg; vitamin D3, 2,000,000 IU; vitamin K3, 1500 mg; biotin, 50 mg; folic acid, 700 mg; nicotinic, 20,000 mg; pantothenic acid, 7000 mg; vitamin B1, 700 mg; vitamin B2, 3500 mg; vitamin B6, 1000 mg; vitamin B12, 7 mg. ^2^ Mineral pre-mix (per kg of pre-mix): zinc sulfate, 4.0 g; iron sulfate, 20 g; manganese sulfate, 5.3 g; copper sulfate, 2.7 g; calcium iodine, 0.34 g; sodium selenite, 70 mg; cobalt sulfate, 70 mg and CaHPO_4_·2H_2_O up to 1 kg. ^3^ Calculated by difference (100 − protein% + lipids% + ash% + crude fiber %). ^4^ Gross energy (GE) was calculated as 5.65, 9.45 and 4.11 kcal/g for protein, lipid and NFE, respectively (NRC, 1993).

**Table 2:** Effect of dietary supplementation with *A. squamosa* leaves extract (ASLE) on growth performance and feed conversion ratio of *O. niloticus* for 60 days.

Items	Dietary *A. squamosa* Leaves Extract (ASLE) Levels (g/kg Diet)					*p* Value		
	0	5	10	15	20	Treatment	Linear	Quadratic
Initial body weight (g)	11.87 ± 0.467	12.50 ± 0.289	12.43 ± 0.318	12.80 ± 0.321	13.07 ± 0.348	0.244	0.37	0.825
Final body weight (g)	42.07 ^d^ ± 0.348	42.73 ^cd^ ± 0.273	45.27 ^c^ ± 0.674	52.67 ^b^ ± 1.453	57.00 ^a^ ± 1.155	0.005	0.0001	0.0001
Weight gain (%)	255.39 ^b^ ± 11.40	242.14 ^b^ ± 5.83	264.28 ^b^ ± 4.14	311.41 ^a^ ± 1.10	336.37 ^a^ ± 2.89	0.001	0.0001	0.0001
Specific growth rate (%)	2.10 ^b^ ± 0.05	2.04 ^b^ ± 0.02	2.14 ^b^ ± 0.01	2.36 ^a^ ± 0.005	2.45 ^a^ ± 0.01	0.003	0.0001	0.0001
Feed intake (g)	52.17 ^c^ ± 0.441	51.17 ^c^ ± 0.441	52.70 ^c^ ± 0.379	56.00 ^b^ ± 0.577	60.00 ^a^ ± 0.577	0.001	0.0001	0.0001
Feed conversion ratio	1.723 ^a^ ± 0.019	1.687 ^a^ ± 0.012	1.600 ^b^ ± 0.006	1.400 ^c^ ± 0.026	1.360 ^c^ ± 0.012	0.08	0.0001	0.0001
Protein efficiency ratio	1.88 ^c^ ± 0.02	1.94 ^bc^ ± 0.01	2.03 ^b^ ± 0.01	2.31 ^a^ ± 0.04	2.37 ^a^ ± 0.02	0.0001	0.0001	0.054
Survival %	100.0 ± 0.00	100.0 ± 0.00	100.0 ± 0.00	100.0 ± 0.00	100.0 ± 0.00	-	-	-

Means with different superscripts are statistically different *p* < 0.05 according to Tukey’s multiple range test.

**Table 3:** Effects of dietary supplementation with *A. squamosa* leaves extract (ASLE) on hematological indices of *O. niloticus* for 60 days.

Items	Dietary *A. squamosa* Leaves Extract (ASLE) Levels (g/kg Diet)					*p* Value		
	0	5	10	15	20	Treatment	Linear	Quadratic
RBCs (10^6^/mm^3^)	2.383 ^b^ ± 0.12	2.477 ^b^ ± 0.09	2.650 ^ab^ ± 0.09	2.760 ^a^ ± 0.07	2.917 ^a^ ± 0.04	0.008	0.001	0.846
Hb (gm/dL)	7.327 ^d^ ± 0.043	7.423 ^cd^ ± 0.038	7.573 ^c^ ± 0.043	7.740 ^b^ ± 0.074	7.970 ^a^ ± 0.038	0.000	0.000	0.154
PCV (%)	21.98 ^d^ ± 0.130	22.27 ^cd^ ± 0.113	22.72 ^c^ ± 0.130	23.22 ^b^ ± 0.221	23.91 ^a^ ± 0.114	0.000	0.000	0.154
MCV (fl)	92.60 ± 3.921	90.13 ± 2.900	85.88 ± 2.323	84.19 ± 1.263	81.99 ± 0.760	0.072	0.006	0.748
MCH (%)	30.87 ± 1.306	30.04 ± 0.966	28.63 ± 0.774	28.06 ± 0.421	27.33 ± 0.253	0.071	0.006	0.747
WBCs (10^3^/mm^3^)	5.357 ^d^ ± 0.030	5.403 ^d^ ± 0.049	5.553 ^c^ ± 0.047	5.877 ^b^ ± 0.043	6.043 ^a^ ± 0.049	0.000	0.000	0.031
Lymphocytes (10^3^/mm^3^)	2.943 ^c^ ± 0.018	2.953 ^c^ ± 0.019	3.017 ^b^ ± 0.020	3.130 ^a^ ± 0.012	3.183 ^a^ ± 0.020	0.000	0.000	0.069
Heterophils (10^3^/mm^3^)	1.433 ^c^ ± 0.018	1.453 ^bc^ ± 0.027	1.503 ^b^ ± 0.015	1.590 ^a^ ± 0.012	1.633 ^a^ ± 0.009	0.000	0.000	0.224
Eosinophils (10^3^/mm^3^)	0.330 ^d^ ± 0.006	0.357 ^d^ ± 0.009	0.393 ^c^ ± 0.015	0.470 ^b^ ± 0.012	0.510 ^a^ ± 0.012	0.000	0.000	0.132
Monocytes (10^3^/mm^3^)	0.650 ^c^ ± 0.006	0.640 ^c^ ± 0.006	0.640 ^c^ ± 0.006	0.687 ^b^ ± 0.009	0.717 ^a^ ± 0.009	0.000	0.000	0.001

Means with different superscripts are statistically different *p* < 0.05 according to Tukey’s multiple range test. RBCs: red blood cells; Hb: hemoglobin; PCV: packed cell volume; MCV: mean corpuscular volume; MCH: mean corpuscular hemoglobin; WBCs: white blood cells.

**Table 4:** Effect of dietary supplementation with *A. squamosa* leaf extract (ASLE) on serum hepatic and renal function, as well as stress indicators of *O. niloticus* for 60 days.

Items	Dietary *A. squamosa* Leaf Extract (ASLE) Level (g/kg Diet)					*p* Value		
	0	5	10	15	20	Treatment	Linear	Quadratic
Total proteins (g/dL)	5.300 ^c^ ± 0.161	5.473 ^c^ ± 0.159	6.183 ^b^ ± 0.073	6.600 ^a^ ± 0.115	6.967 ^a^ ± 0.073	0.001	0.001	0.843
Albumin (g/dL)	2.320 ^d^ ± 0.062	2.290 ^d^ ± 0.038	2.640 ^c^ ± 0.049	2.883 ^b^ ± 0.073	3.090 ^a^ ± 0.042	0.001	0.001	0.102
Globulin (g/dL)	2.980 ^c^ ± 0.117	3.183 ^c^ ± 0.148	3.543 ^b^ ± 0.023	3.717 ^ab^ ± 0.044	3.877 ^a^ ± 0.032	0.001	0.001	0.428
ALT (U/L)	12.21 ^a^ ± 0.653	12.19 ^a^ ± 0.641	11.08 ^ab^ ± 0.159	10.78 ^ab^ ± 0.280	10.12 ^b^ ± 0.093	0.026	0.002	0.773
AST (U/L)	27.70 ^a^ ± 0.321	27.63 ^a^ ± 0.291	27.02 ^ab^ ± 0.073	26.85 ^b^ ± 0.132	26.72 ^b^ ± 0.117	0.023	0.002	0.698
ALP (IU/L)	24.27 ^a^ ± 0.088	24.19 ^a^ ± 0.058	24.08 ^ab^ ± 0.060	23.87 ^b^ ± 0.104	23.61 ^c^ ± 0.059	0.001	0.001	0.132
Urea (mg/dL)	2.767 ^a^ ± 0.027	2.760 ^a^ ± 0.023	2.687 ^ab^ ± 0.032	2.590 ^b^ ± 0.026	2.220 ^c^ ± 0.057	0.001	0.001	0.001
Creatinine (mg/dL)	0.447 ^a^ ± 0.012	0.440 ^a^ ± 0.006	0.423 ^a^ ± 0.009	0.357 ^b^ ± 0.015	0.317 ^c^ ± 0.012	0.001	0.001	0.018
Cortisol (ng/L)	53.53 ^a^ ± 0.906	52.27 ^a^ ± 0.657	51.40 ^a^ ± 0.737	46.27 ^b^ ± 1.105	43.17 ^c^ ± 0.441	0.001	0.001	0.024
Glucose (mg/dL)	75.43 ^a^ ± 1.260	74.53 ^a^ ± 1.017	73.07 ^a^ ± 1.090	68.40 ^b^ ± 1.127	63.33 ^c^ ± 0.982	0.001	0.001	0.019

Means with different superscripts are statistically different *p* < 0.05 according to Tukey’s multiple range test. ALT: Alanine aminotransferase. AST: Aspartate aminotransferase. ALP: Alkaline phosphatase.

**Table 5:** Effect of dietary supplementation with *A. squamosa* leaf extract (ASLE) on the serum and liver homogenate oxidative/anti-oxidative status of *Oreochromis niloticus* for 60 days, as well as its mortality rate after cold challenge for 14 days.

Items		Dietary *A. squamosa* Leaf Extract (ASLE) Levels (g/kg Diet)					*p* Value		
		0	5	10	15	20	Treatment	Linear	Quadratic
Serum	TAC (mM/L)	1.623 ^c^ ± 0.050	1.847 ^c^ ± 0.032	2.070 ^c^ ± 0.117	3.160 ^b^ ± 0.181	4.350 ^a^ ± 0.465	0.001	0.001	0.009
	CAT (U/L)	69.60 ^d^ ± 1.900	74.73 ^cd^ ± 0.561	77.90 ^c^ ± 0.693	89.83 ^b^ ± 2.429	97.90 ^a^ ± 1.908	0.001	0.001	0.041
	SOD (U/mL)	6.100 ^c^ ± 0.569	6.833 ^c^ ± 0.504	7.500 ^c^ ± 0.404	9.567 ^b^ ± 0.578	13.50 ^a^ ± 0.866	0.001	0.001	0.006
	GSH (μmol/mL)	11.43 ^c^ ± 0.517	12.70 ^c^ ± 0.473	13.27 ^c^ ± 0.318	15.63 ^b^ ± 0.593	18.57 ^a^ ± 0.809	0.001	0.001	0.036
	MDA (nmol/mL)	13.23 ^a^ ± 0.722	12.33 ^ab^ ± 0.088	11.87 ^b^ ± 0.203	9.833 ^c^ ± 0.145	9.300 ^c^ ± 0.153	0.001	0.001	0.539
	MPO (U/L)	63.23 ^a^ ± 0.536	62.17 ^a^ ± 0.176	61.70 ^a^ ± 0.379	53.03 ^b^ ± 1.415	45.83 ^c^ ± 1.878	0.001	0.001	0.001
Liver homogenate	CAT (U/g tissue)	1.76 ^d^ ± 0.04	1.85 ^d^ ± 0.04	1.98 ^c^ ± 0.03	2.16 ^b^ ± 0.03	2.35 ^a^ ± 0.03	0.001	0.001	0.093
	SOD (U/g tissue)	4.26 ^e^ ± 0.04	4.40 ^d^ ± 0.03	4.59 ^c^ ± 0.04	4.82 ^b^ ± 0.05	5.07 ^a^ ± 0.05	0.001	0.001	0.126
	MDA (nmol/g tissue)	18.47 ^a^ ± 0.93	16.40 ^b^ ± 0.72	14.27 ^c^ ± 0.62	12.6 ^d^ ± 0.46	11.13 ^e^ ± 0.19	0.001	0.001	0.498
Post-cold challenge mortality %		6.66 ± 6.66	0.00 ± 0.00	0.00 ± 0.00	0.00 ± 0.00	0.00 ± 0.00	-	-	-

Means with different superscripts are statistically different *p* < 0.05 according to Tukey’s multiple range test. TAC; total antioxidant capacity; SOD: superoxide dismutase; CAT: catalase; GSH: reduced glutathione; MDA: malondialdehyde; MPO: myeloperoxidase.

## Data Availability

On request, the authors will supply all necessary data.
